# Interaction between non-executive and executive directors in English National Health Service trust boards: an observational study

**DOI:** 10.1186/s12913-015-1127-2

**Published:** 2015-10-15

**Authors:** Rod Sheaff, Ruth Endacott, Ray Jones, Val Woodward

**Affiliations:** School of Government, Plymouth University, Portland Villas, Drake Circus, Plymouth, PL4 8AA UK; School of Nursing & Midwifery, Plymouth University, Portland Villas, Drake Circus, Plymouth, PL4 8AA UK; School of Nursing & Midwifery, Monash University, Melbourne, Australia

**Keywords:** Corporate governance, Clinical governance, Non-executive directors, Interaction between directors, Perlocution

## Abstract

**Background:**

National Health Service (NHS) trusts, which provide the majority of hospital and community health services to the English NHS, are increasingly adopting a ‘public firm’ model with a board consisting of executive directors who are trust employees and external non-executives chosen for their experience in a range of areas such as finance, health care and management. In this paper we compare the non-executive directors’ roles and interests in, and contributions to, NHS trust boards’ governance activities with those of executive directors; and examine non-executive directors’ approach to their role in board meetings.

**Methods:**

Non-participant observations of three successive trust board meetings in eight NHS trusts (primary care trusts, foundation trusts and self-governing (non-foundation) trusts) in England in 2008–9. The observational data were analysed inductively to yield categories of behaviour reflecting the perlocutionary types of intervention which non-executive directors made in trust meetings.

**Results:**

The observational data revealed six main perlocutionary types of questioning tactic used by non-executive directors to executive directors: supportive; lesson-seeking; diagnostic; options assessment; strategy seeking; and requesting further work. Non-executive board members’ behaviours in holding the executive team to account at board meetings were variable. Non-executive directors were likely to contribute to finance-related discussions which suggests that they did see financial challenge as a key component of their role.

**Conclusions:**

The pattern of behaviours was more indicative of an active, strategic approach to governance than of passive monitoring or ‘rubber-stamping’. Nevertheless, additional means of maintaining public accountability of NHS trusts may also be required.

## Background

As the state in many European countries is minimised in favour of the provision of publicly-funded services, if not by private providers then by ‘public firms’ modelled upon them, the question of how to maintain the public accountability of these services becomes more pressing. This paper examines how board members of English National Health Service (NHS) organisations during 2008–9 behaved in that role.

### Boards and governance

Boards of directors are intended, both in healthcare and in other sectors, to exercise corporate governance over public and private corporations [1]. Researchers have tended to apply principal-agent theory when analysing corporate boards’ relationship to managers and so, indirectly, to workers [2, 3]. That theory predicts a potential ‘principal-agent problem’ i.e., the risk that managers will manage their organisation self-interestedly rather than in the interests of those whom the board represents [4] or, doubting the legitimacy of the board’s authority, informally resist the board [5]; all of which is taken to imply the need for means to align artificially the executive directors’ normative beliefs and interests with those of the non-executive directors who represent the non-managerial stakeholders in the board. In corporate boards the latter are predominantly financial interests (shareholders, investors); in public organisations’ boards, the state’s interests; and in third sector organisations’ boards either the general public or more ‘diverse’, specific stakeholders’ interests [6, 7]. For corporations, therefore, one means for pre-empting this anticipated ‘principal-agent’ problem has been to pay top managers partly in shares or share options [8, 9]. However this method may have the perverse effect of incentivising managers to focus their efforts on raising share prices in the short-term. It is anyway irrelevant to non-corporate settings, where other means are required for aligning managers’ interests with those of the ‘stakeholders’ whom the board represents. These means include:

1. A transactional approach: the board and managers negotiate a modus vivendi which appeals to the intrinsic, not only the financial, motivations of either party [5].
2. Purposive selection of board members, both individually and in terms of the mix of members in the board as a whole.
3. Educating and training non-executive board members in the behaviours required to control their organisation’s strategic direction and outcomes [10], exercising this influence vicariously through the executive directors and other managers. That behaviour includes questioning, challenging and supporting the executive directors [11],
4. Board scrutiny of the work of executive directors (EDs), other managers and staff [12].

The third and fourth rely upon what topics the non-executive directors (NEDs) take an interest in, who they consult about them, and what contributions the NEDs make to the board’s work both within and outside of the confines of board meetings [13].

Empirical studies – which are mostly of US organisations - present a mixed picture of how far these methods work as intended. Board composition and credibility appears to influence the organisation’s ability to obtain resources externally, particularly when the organisation is contractually accountable to public bodies which commission (sub-contract) it [14]. A cohesive board, and one whose members participate in board educational activities, appears to help its organisation function efficiently, a discordant board the opposite [15]. Three antecedents of board task performance are diversity of board membership, commitment (‘stewardship’ [16]) and critical debate [2]. Against this, in 2008 a UK Government report on corporate banking failures found that the essential ‘challenge’ step in board decision-making processes was often missing, and that the role of NEDs should be more than a ‘rubber-stamping’ exercise, with the appointment of NEDs who are prepared to focus on high level risk issues [16]. Although their boards do occupy themselves mainly with financial oversight and policy making, many US non-profit organisations display a ‘board gap … the difference between board performance and the expectation of boards’ [14]. Having a chief executive as a voting board member tended if anything to reduce board accountability [17]. NEDs on UK corporate boards most strongly promote board accountability when they are ‘engaged but non-executive’, ‘challenging but supportive’ of managers and ‘independent but involved’ [11]. In summary, the behaviours through which board members make managers and workers accountable appear to fall within three main approaches:

1. selecting topics of interest for scrutiny and discussion at board meetings, but this is done mostly outside board meetings and by the board chair and secretary (or the equivalents).
2. Initiating strategies to influence the organisation’s future work, not just responding passively to the executives’ proposals.
3. attributing responsibility to specific executives for specific activities.

All three strategies have implications for who the non-executives consult, and about what topics, both inside and outside board meetings.

These behaviours are accomplished through speech acts: the use of speech for practical purposes. As our analytic framework we therefore adopted Austin’s account of the three main elements of speech acts: locution (cognitive content); illocution (the kind of act performed by the speech act); and perlocution (the behavioural, practical response which the speaker is trying to produce) [18]. We used Austin’s theory because it is a form of discourse analysis that links the content of a speech act (in this case, what board members said; what Austin calls the ‘locution’ of a speech act) with its intended effects on others’ behaviour (its ‘perlocution’) both in its immediate setting (i.e., board meetings) and beyond (i.e., in the rest of the organisation). In this respect Austin’s approach contrasts with, say, cognitive frame [19], Leximancer [20] and logic [21] analysis methods of discourse analysis. For this study, the perlocutionary effects of directors’ behaviour were most relevant because we were interested in how board members’ behaviour within board meetings acts – or fails to – as a mechanism for exercising governance by means of persuading, negotiating with [5] or in some other way directing the executive directors present at a board meeting to take certain prescribed actions afterwards outside the meeting, in the rest of the organisation.

### Trusts and boards in the English NHS

General practice apart, NHS organisations are mostly structured as ‘trusts’, of which three main varieties existed at the time of this research (2008–9). Primary care trusts (PCTs) were responsible for coordinating general practices and for commissioning (purchasing) secondary care, community and mental health services. NHS trusts (with no qualifying adjective) were the usual form of organisation for acute hospitals, mental health services and, increasingly, community health services. Foundation trusts (FTs), described below and also known as Self-Governing Trusts (SGT), were another form of provider organisation. Governance over all three types of trust was exercised by a board of executive and non-executive members [22]. Across all NHS Boards, 42.4 % of members were NEDs in 2010 [23].

NHS trusts were set up at the start of the 1990s. Their boards of directors consist of EDs who are employees of the organisation and hold senior positions in the Trusts, and NEDs who are not directly employed, but are appointed to sit on boards because of their expertise in areas such as finance, management and clinical matters [24–27]. NEDs are expected to challenge and clarify items with EDs in order to provide governance and stewardship within the organisation [28]. NHS boards of directors are held accountable to higher NHS bodies, at the time of this research strategic health authorities (SHAs), for their actions. They have to produce annual reports and audited accounts and hold an annual general meeting [29].

Similar structures were created in PCTs. To advise its board each PCT had a professional executive committee of general practitioners (GPs). Initially PCTs line-managed primary care services other than those provided by general practices, but from 2004 these services were transferred to separate organisations commissioned by the PCTs, mostly NHS trusts but occasionally social enterprises. At the time of this research PCTs both commissioned this range of services and, in some places, still directly managed a residuum of community health services. Meantime policy-makers were pressing NHS Trusts to convert into Foundation Trusts (FTs). Created in 2006, this new class of trust was set up to increase local accountability with more freedom to invest and disinvest [30]. FTs are responsible to Monitor, an NHS regulatory authority, for their performance and market behaviour. In addition their boards of directors are responsible to a board of governors, selected from the public served by that organisation.

Apparently NHS boards too display a ‘board gap’ at times. Ferlie et al. [22] found that English NHS boards tended to endorse managers’ decisions rather than make their own, although NHS boards have been re-structured and re-populated several times since then. A commissioning board for mental health services was found to serve not so much a decision-making function as being a place of ritual ‘where participants tell narratives about who they are collectively, sustain culture, organize shared emotions, and conciliate over social relations in conflicts … to maintain organizational cohesion above all’ [31]. A review of governance arrangements in NHS FTs indicated that the additional structures in place, allowing patient, public and staff input, needed further development to demonstrate value [32].

#### Study aims and research questions

These findings therefore raise the questions of whether NHS board NEDs do indeed steer, even initiate, their organisation’s strategy, monitor its activity and so behave in board meetings as to enable the boards to exercise control and scrutiny over the organisations whose governance they are responsible for. In this study we therefore aimed to explore further how the role of NEDs in NHS boards differs from that of EDs, how the two kinds of director interact in the boards, and what the implications for the governance of NHS organisations might be. Our research questions (RQs) were:

1. What practical responses were NEDs trying to elicit, at board meetings, from the NEDs and how did NEDs contribute to the meetings?
2. What governance strategies do the above patterns of behaviour appear to represent?
3. Which topics were NEDs (and EDs) most interested in?

There are few other studies of NHS board roles, interests and relationships. This study therefore adds to the evidence about the governance and processes of stewardship in NHS board of directors and interaction between those in executive and non-executive roles. So far as the NHS is concerned, the Francis enquiry [33] exposed the weighty consequences of failing to ensure the public accountability of healthcare providers. As healthcare reforms, both in England and other countries make even publicly-owned healthcare providers increasingly autonomous, these questions have increasing and wider importance.

## Methods

### Research design

We combined a structured observation of board meetings and a cross-sectional sample survey of board members. This paper concentrates on the former. (Details of the survey are available on request from the authors.) We observed the meetings for each of eight boards, categorising the behaviours that we saw board members use by applying categories which we had developed from those used in existing empirical studies [31, 34] and from the theory of speech acts. In applying these categories, and refining them by doing so, we formed some initial conclusions about board members’ modes of behaviour, the topics of board discussion, and which board members tended to take the lead on particular topics.

#### Sample and settings

A mix of PCTs, acute FTs and acute SGTs were included in both parts of the study. We selected a sample of eight English NHS trust boards for observation, in order to collect data suitable for qualitative analysis. This was a theoretically-based selection. We assumed that boards with different functions within the NHS might discuss different topics, and that relationships between ED and non-ED members, and the ways in which meetings were conducted, might correspondingly differ. Of the eight boards, four (PCTs) were predominantly commissioners, and four providers of care. (The latter were acute care trusts, two of them SGTs and two FTs.) We were able to observe both the open (part 1) and private (part 2) part of the board meetings in five organisations. In the remaining three trusts, part 1 only was observed; two provider trusts refused access to part 2 and one commissioning trust had not decided by the time the study ended. Table 1 characterises the eight trusts whose board meetings we observed.

**Table 1:** Characteristics of trusts whose board meetings were observed

Dominant Role	Site	Characteristics	Part 2 observed [Y/N]
Commissioner	PCT 1	Inner-city, deprived area, high ethnic minority population	N
	PCT 2	Rural area serving a large area and population	Y
	PCT 3	Mixed rural and urban, largely affluent area	Y
	PCT 4	Inner city, deprived area, high ethnic minority population	Y
Provider	SGT 1	Large teaching hospital, city setting	N
	SGT 2	Teaching hospital, mixed urban and rural setting	N
	FT 1	District general hospital, largely rural population	Y
	FT 2	District general hospital in small city, mixed urban and rural population	Y

*PCT* primary care trust, *SGT* self-governing acute trust, *FT* acute foundation trust

All types of trust were undergoing transition at the time of the study (2008–9). PCTs had recently undergone mergers and become essentially commissioning organisations, not providers. The SGTs were considering applying to become FTs, and the FTs were still in the early stages of adjusting to their new status and of being accountable to both a board of directors and a board of governors. We used a survey (not reported here) to assess whether findings from the sites described in Table 1 were likely to have external validity, which they were. In brief, the ratio of EDs to NEDs in the observational sample was similar to the ratio among all NHS Board members [23].

### Data collection

Three consecutive meetings were observed in each of the eight NHS trust boards i.e., 24 observations totalling over 80 h. Methods of designing the observation, to include construction of the observation schedule, have been previously reported [34]. Briefly, the observation schedule recorded the ‘presenting problem’ (taken to include any practical proposals or action points) and who raised it. Besides directors’ behaviours, we recorded the main points of the debate, who responded, and how, to the ‘presenting problem’ raised by a NED, and what decision the board finally made. There were also sections for other qualitative observational notes and quotations. The schedule and its coding sheet were pilot tested for reliability before use. From publicly available biographical information on NHS trust websites we also ascertained the occupational background of the board members we observed.

### Analyses

We analysed the observational data inductively. We classified directors’ behaviour according to the perlocution [35] of their questions and statements in board meetings, i.e., in terms of what type of practical response that the speaker (NED) was apparently trying to elicit from the board members who were being addressed (e.g., getting an ED to agree to alter a proposed course of action). We took this approach because a perlocutionary categorisation tells us:

1. the range of practices (e.g., option assessment, seeking information etc.) through which NEDs tried to influence EDs’ managerial work; and
2. which behaviours within this repertiore NEDs most heavily relied upon at Board meetings

Also a perlocutionary classification offers a framework for:

3. systematically comparing how NEDs’ use of these behaviours varied between different organisational types; and
4. imputing what the NEDs assumed were legitimate practical demands to make of the EDs; hence describe what, at minimum, NEDs thought their role involved.

A perlocutionary classification captures the practical content (locution) of a speech act, in this case *what* the EDs were being asked to do. It tells us how, within Board meetings, NEDs exercised their role through speech acts. It does not necessarily capture NEDs’ tone of voice, attitudes, whether an NED was trying to persuade, intimidate, cajole etc., nor impute to the speaker any implicit, unspoken perlocution behind the overtly stated one. Insofar as they reflected hidden interpersonal agendas, We observed whether those being addressed appeared to understand, and whether they appeared to comply or made counter-responses.

Two researchers categorised the directors’ behaviours from field notes made during the 24 periods of non-participant observation of board meetings, devising new categories until saturation (no more appeared necessary for classifying the data), then de-duplicating this initial set of categories to yield an irreducible set of six. Each category was defined, and differentiated, in terms of what practical response the director appeared to be seeking from the board and managers, i.e., to:

1. encourage existing activities and decisions (‘supportive’ behaviour).
2. suggest practical steps to prevent the recurrence of past problems (‘drawing [practical] lessons’).
3. analyse what had caused such problems or, conversely, successes (‘diagnostic’ behaviour):
4. evaluate which strategy was preferable (‘option assessment’)
5. elicit suggestions about how to develop the organisation’s future work (‘strategy-seeking’)
6. initiate the production of information about the organisation’s current or recent activities (‘seeking fuller reports’).

Items (3) and (6) respectively concerned explanations and factual information about the current and recent past activity. Items (2) and (5) elicited proposals for action (responding to past events and shaping the organisation’s future activity), and items (1) and (4) elicited normative judgements (respectively about the present and future). The empirical distribution of behaviours across these categories therefore reveals whether directors attended more in practice to retrospective (e.g., monitoring) or prospective (e.g., strategy) issues, and more to descriptive, analytic (e.g., diagnostic) or normative (e.g., legitimating past or choosing future activity) questions.

Some utterances might be interpreted as having more than one of the perlocutions listed above (e.g., an ostensibly supportive comment such as *‘How have the Outpatient clinics coped with the increase in activity?’* might also be a veiled request to diagnose the trust’s current activity to assess whether the increase was sustainable). In such cases we coded each utterance according to what the researchers agreed appeared to be its primary perlocution, given its context and the practical implications that those at the board meeting took to follow from it. By ignoring what might be called secondary perlocutions, this approach tended to under-record the number of perlocutionary acts occurring in board meetings, so in that sense be incomplete.

What would it mean for a Board, even an organisation, if its NEDs tended especially to use certain of these perlocutions rather than others? Above we noted that, agenda-setting apart, the two main approaches by which NEDs might influence other Board members and through them the rest of their organisation were:

1. proposing strategies for their organisation’s future work; and
2. holding EDs and other staff to account for its current and past work.

To see NEDs relying heavily on seeking practical lessons, option assessment and strategy-seeking would suggest a predilection for the first of these. To see them rely heavily on diagnosis of past failures or successes, seeking further reports and, if applicable, supportive behaviour would suggest they preferred the second approach, emphasising the monitoring of what their organisation did. Passive, ‘rubber-stamping’ NEDs would *themselves* make little use of diagnostic behaviour and option-appraisal but they, and NEDs who wished to avoid discord, would make much use of supportive behaviours. NEDs who focused on risky strategic decisions would rely most upon diagnostic behaviour, lesson-seeking and option appraisal. In short, different selections of perlocutions would constitute different strategies by which NEDs exercised governance through their Board, with correspondingly different balances of emphasis on recent, current or future activity. Insofar as NEDs’ influence extended beyond the Board, one would expect the wider management style of the organisation then to display similar patterns.

### Research ethics and governance

The NHS Research Ethics Service (reference number 08/H0104/5) and the Plymouth University Faculty of Health Research Ethics Committee granted approval; approval was also gained from individual trust research and development officers under the NHS research governance framework. We obtained written consent for the observations from members of NHS Trusts. As a condition of ethical approval, informants and study sites are pseudonymised.

## Results

The observational analysis suggested that NEDs used six main types of questioning tactic. Table 2 gives examples.

**Table 2:** Types of NED perlocution observed in board meetings

Type 1 Supportive
*I am impressed with this report and the extent of working with partners*. (Site D, PCT).
*Are you happy with the 4 h target?* [Board setting criteria for pilot work in the new Minor Injuries Unit] (Site A, PCT).
*How are the problems with the NHS IT system affecting the staff at grass-roots level?* (Site E, FT).
*Are you content with the length of time between an incident and the report; is it good enough?* (Site F, FT).
Type 2 Lesson-seeking
*How have we managed to reduce both long term and short-term sickness?* (Site H, SGT).
*Do we know why our C. Diff rates have improved so much?* (Site E, FT)
*The success of the sexual health model is to be applauded; is there a model here that we can also use for immunisation?* (Site C, PCT).
Type 3 Diagnostic
*Why are we not achieving targets for out of hours diabetes services; is the target unrealistic?* (Site B, PCT).
*Why is COPD not included in the top ten* [World Class Commissioning] *priorities?* (Site D, PCT).
*It says here that we’re running at 148 % capacity: how?* (Site G, SGT).
*Why are almost 50 % patients waiting more than 31 days?* (Site A, PCT)
*Why are we focusing on hips and knees* [in reducing waiting lists]? (Site E, FT).
*Communication* [with patients whose appointments were cancelled] *is a weak area and needs improving.* (Site G, SGT)
Type 4 Option Assessment
*Will the new informatics strategy improve co*-*ordination of diabetes management*? (Site B, PCT).
W*hat are the complaints about? We’re far more interested in the nature of the complaints; we need more detail on this rather than the process to resolve them.* (Trust G, SGT).
*What are the additional costs for pre-op screening of all patients* [for MRSA]? (Site H, SGT).
*The risks and staffing are quite different for home and hospital births; are the tariffs* [i.e., payments to the hospital] *different?* (Site E, FT).
Type 5 Seeking Strategy
*How will we know that changes* [resulting from a Health Care Commission report on one of the provider trusts] *are being sustained*? (Site B, PCT)
*Who are we consulting with* [about regionalisation of stroke and trauma services]? (Site C, PCT).
*How will we review this* [Health Care Commission] *rating during the year to ensure we’re not in the same situation again?* (Site F, FT).
*How can we take this* [problem with inpatient and outpatient waiting times] *forward in relation to our Foundation Trust application?* (Site H, SGT).
*When will we see improvement in the privacy and dignity performance indicators?* (Trust D, PCT).
Type 6 Requesting Fuller Reports
*We can’t see what the targets are and what the current baseline is; this needs different presentation.* (Site A, PCT).
*Can you please provide a separate report on orthopaedics each time and add it to the exception reporting.* (Site F, FT).

Supportive comments (see Table 2) acknowledged - in one case to the extent of being congratulatory, and in others by means of leading questions – that the organisation was functioning as the board desired *(e.g., I am impressed with this report and the extent of working with partners.*), endorsing them, encouraging them to continue and re-asserting that board decisions, or the actions taken to implement them, had been sound. Lesson-seeking consisted of assertions or questions about the underlying reasons for the kinds of events that might be the subject of the supportive comments *(e.g., do we know why CDiff* [Clostridium Difficile infection] *rates have improved so much*?). Diagnostic comments and questions *(e.g., ‘Why are we focusing on hips and knees*?’) were used to explain or question why apparent problems, failures or unforeseen events had occurred, and so challenge EDs to account for their decisions. NEDs used strategy-seeking questions to challenge EDs to explain and substantiate how they would achieve objectives set by, or externally imposed on, the board (e.g., *How will you get up to target*?). The focus or content of strategy seeking questions therefore depended on the type of trust. NEDs’ strategy-seeking questions (e.g., *What is the average length of stay for all patients*?) were typically seeking to discover:

1. how patient pathways were working or might be expected to change if the board took a particular decision,
2. the extent of compliance with standards of care,
3. the cost implications of clinical practices.

Unlike supportive comments or questions, strategy seeking questions challenged EDs to justify their assertions or decisions. Requests for further reports (e.g., *Can you examine why we have an increase in DNAs* [patients who missed outpatient appointments]?) usually supported the board’s scrutiny role. Their content reflected the type of trust. NEDs on PCT boards were more likely to ask questions about the PCT’s commissioning activities whereas NEDs for the provider trusts were more focused on clinical outcomes. Categories (1) to (6) each related a director’s behaviour to a local context, although what that context was, hence the substance of each suggestion, demand etc., obviously depended on each organisation’s particular circumstances at the time and the meeting agenda.

In the observed meetings, drawing lessons was NEDs’ least frequent intervention, strategy-seeking the most frequent NED contribution to board meetings (Table 3).

**Table 3:** Types and numbers (percentage) of Non-Executive Director intervention observed during board meetings

	PCT				FT		SGT		ALL
	A	B	C	D	E	F	G	H	
Supportive comments	5(11 %)	0(0 %)	0(0 %)	6(25 %)	7(16 %)	1(4 %)	3(8 %)	1(3 %)	23(9 %)
Lessons learnt	2(4 %)	0(0 %)	3(9 %)	1(3 %)	3(7 %)	0(0 %)	0(0 %)	2(6 %)	11(4 %)
Contextual comments, Questions	8(18 %)	7(27 %)	3(9 %)	4(17 %)	10(23 %)	5(20 %)	7(18 %)	6(18 %)	50(19 %)
Option assessment	14(32 %)	8(31 %)	7(23 %)	5(21 %)	15(36 %)	8(32 %)	7(18 %)	9(27 %)	73(27 %)
Strategy seeking	11(24 %)	7(27 %)	10(31 %)	4(17 %)	7(16 %)	10(40 %)	9(24 %)	4(12 %)	62(23 %)
Requesting further work	5(11 %)	4(15 %)	9(28 %)	4(17 %)	1(2 %)	1(4 %)	12(32 %)	11(34 %)	47(18 %)
TOTALS (100 %)	45	26	32	24	43	25	38	33	266

Table 3 shows that NEDs’ behaviour was on balance more suggestive of the approach of exercising governance by proposing strategies for their organisation’s future work than that of attributing responsibilities to executives for its current and past work. The former (option appraisal, strategy-seeking) accounted for half of the NEDs’ interventions. The general qualitative pattern was that NEDs saw their roles in meetings as being to interrogate what the organisation’s managers (including the EDs, but not only they) were doing, to make feedback about organisational (but not usually personal) performance, and to select the main directions for future activity. EDs saw their roles in meetings partly as responding to the feedback and requests for practical proposals, also as helping to frame the range of strategies and feedback issues that NEDs would interrogate. However, we observed variations in this general pattern. Some NEDs contributed little to meetings and asked few questions, whilst others frequently asked questions and asked EDs to explain further or undertake further work in a variety of areas. In particular the extent of discussion of clinical matters depended on the ways in which NEDs questioned EDs. The lengths of meetings varied considerably (45 min to 5 h, mean 3 h 20 min) and were generally longer where NEDs used a variety of questioning tactics that generated discussion and debate. The level of debate and discussion in board meetings varied according to the questioning tactics and willingness of the NEDs to question EDs and Chief Executives.

We observed three dimensions to this dynamic. One was how the NED role was set up at organisational level, for example what part the NEDs played in governance structures outside the Board itself. NEDs’ ability and freedom to question the EDs appeared to depend on what further roles, besides board membership, the NED had in the organisational structure and upon the NED’s personal ability and willingness to question EDs. NEDs’ precise roles varied across trusts. Thirty-four of the 135 NEDs identified further roles that they held. Nineteen were chair and eight vice chair of the board, and seven were chair of the audit committee. Most were chairing committees, for example the clinical governance committee or audit committee, depending on their skills. At one trust NEDs regularly visited wards and other clinical areas and had a regular agenda slot to feed back what they found. Whilst we limited our observations to the board meetings, it was evident, and not surprising, that NEDs with these additional roles played a more active role in discussions related to their governance role. Nevertheless these NEDs still retained a ‘non-executive’ attitude in these discussions in that they did not become defensive of Trust actions in their discussions at the board. There was no obvious relationship between the extent and type of board meeting contribution and the occupational background of the NED. Neither did the length of time served on the board (or, in the case of newly merged trusts, predecessor bodies) appear to influence the contribution made by individual NEDs.

Second, NEDs’ interests in particular areas of questioning tended to wax and wane depending on the context in which they were discussed. For example a patient complaint could suddenly evoke high levels of interest if it reflected poorly on the organisation. Similarly, if there were a staffing crisis affecting care-giving or achievement of targets, interest in human resources moved up the scale. Clinical ethics and governance attracted more interest if there was an ethical dilemma (such as balancing the costs and efficacy of medications) or issue with the implementation of evidence-based practice. Also the agenda usually constrained what NEDs would ask about.

Third, we observed variations in how actively or passively EDs responded to NED requests, questions and comments. At one pole, ED responses were passively compliant, for instance in minuting the NED’s request (e.g., to amend board minutes to record difficulties as well as successes in meeting control of infection targets) or in agreeing with what the NED had said (e.g., about the financial situation, staff morale). More actively, EDs agreed to chase up promised actions which had not taken place or bring the matter forward to a subsequent board meeting. In a few cases, EDs provided then and there missing information whose absence an NED had pointed out. EDs also undertook to hold further discussions outside the board meeting (e.g., to correct alleged inaccuracies in reports about capital grants) and to guarantee NED involvement in board sub-committees (e.g., for audit, remuneration). This was the nearest that EDs came to making counter-responses to what the NEDs said. We did not observe any occasion on which an ED overtly challenged the NED’s intervention or showed any outward sign of non-compliance. NEDs were prepared to press EDs to substantiate what the EDs were stating or proposing, and sometimes insisted on being kept informed of (even involved in) work directly arising from Board decisions. We observed no occasions on which NEDs simply over-ruled the EDs.

The main substantive foci of board discussion were the overall performance and competence of the management of the trust (‘corporate governance’), the management of clinical quality (‘clinical governance’) and finance. In the SGTs, which were preparing their applications for FT status, NEDs were more likely to request further reports in the Part 1 (public) meetings. Much of the discussion in the private part of PCT board meetings related to their dual role at that time of commissioner and provider and, in particular, to the purpose of NED participation in managerial sub-committees. There was also considerable discussion of the NEDs’ role during the Part 2 (i.e., private) component of board meetings:

*The non-executive directors are being used in too operational a manner; the NED role should be to challenge the executive directors* (NED, PCT 1)*.*

*We need maximum engagement between the Non-Executive Directors and the Executive Directors* (Chair, PCT 4)

*We need to ensure that the Non-Executive Directors are kept in the loop* [regarding media interest in a serious untoward incident] (Chief Executive Officer, FT 2)

*The NED should ensure that appropriate advice has been taken* (Chair, PCT 1)

NEDs’ ability, willingness and freedom to question EDs and hold them to account appeared to vary across organisations, depending on both NEDs’ personal characteristics and the manner in which the role was established by the chair.

We also observed that EDs intervened more often to discuss concrete, practical aspects of service provision: service design, evidence base for practice and referral rates and volumes, i.e., what might be described as the more technical aspects of management, both financial and ‘real side’ (e.g., activity rates), than NEDs. NED interventions gave weight to broader service outcomes such as patient feedback and complaints, relationships with stakeholders, clinical ethics and clinical outcomes. Otherwise NED and ED patterns of intervention did not appear to differ much. Taken together, NEDS and EDs showed more interest in retrospective (outcome monitoring) and diagnostic issues than NHS policy agendas, although service design was often discussed. EDs were more likely to contribute about topics that were subject to national performance targets, especially those which combined clinical and policy issues, for example, service design and standards. These topics have potential implications for risk management (hence managers’ accountability or hospital liability), but for other target-driven topics - clinical outcomes, referral rates and activity levels - EDs and NEDs equally contributed to board discussions and decisions. We observed that NEDs did nevertheless contribute actively to finance-related discussions. This suggests that NEDs saw financial questioning as a key component of their role (regardless of whether they were personally interested in the subject [34]).

## Discussion and conclusions

### Qualifications to findings

Certain clear patterns and contrasts in ED and NED behaviour became evident from the interactions observed across the 80 h of board meetings. The observations suggest what directors’ role was in practice, irrespective of directors’ attitudes or normative beliefs about what their role *ought* to be. One might impute the latter from their speech acts, but such questions are better researched by depth-interviews. Conceivably NEDs’ assumptions and attitudes on these points might in turn partly reflect the wider NHS policy and institutional contexts. These matters merit further research. The adjunctive survey suggested that the pattern of interaction observed across the 80 h of board meetings has wider, external validity. We did not study what practical consequences for the rest of the study organisations, in particular for their organisational behaviour and transparency (accountability), resulted from NED activities at Board meetings. A perlocutionary classification of NEDs’ verbal behaviours in Board meetings cannot accomplish that, and did not aim to. For that would require another substantial implementation study, looking within rather than across organisations, and investigating how far the approaches to governance (e.g., strategy-setting *versus* monitoring *versus* rubber-stamping) represented by NEDs’ perlocutionary behaviour within Board meetings were carried through into managerial practice and styles outside Board meetings, and to what effect. The relationships between board characteristics and an organisation’s performance are mediated through many complex factors - workforce characteristics, organisational structure and external resource dependencies, to name but three.

NHS organisations from other UK countries were not included due to their differing nature following devolution of power for the NHS and thus differing NHS structures. Since this research was undertaken the English NHS has been ‘reformed’ yet again. However the governing bodies of Clinical Commissioning Groups (CCGs), which in 2013 replaced PCTs, are still required to include ‘independent lay members’ and comply with similar ‘Principles of Good Governance’ [36] as apply to NHS Foundation Trusts, which continue as before. NHS trust boards remain structured, populated and centrally accountable (now to NHS England rather than SHAs), essentially as described above. Our findings remain relevant to the present-day NHS, indeed are likely to become increasingly so as NHS trusts are encouraged to become more like ‘public firms’.

### Empirical findings

In summary, NEDs used six main perlocutionary types of intervention in board meetings: supportive comment; lesson-seeking; diagnostic questions or comment; option assessment; strategy-seeking; and seeking further work. They relied most on strategy-seeking and least on lesson-seeking. In Board meetings they were active in financial monitoring. EDs’ interventions tended to concentrate on detailed operational questions (referral rates, service design, evidence-based practice). Our findings suggest that NEDs did intervene to scrutinise and control the main activities for which their organisations were responsible, foci which varied between boards, reflecting current policy priorities and their organisations’ role within the NHS. NEDs showed greatest interest in the topics of patient experience (clinical and non-clinical), with the exception of evidence-based practice, and least in the more purely managerial topics. Non-executive members of NHS boards took interest in what policy makers regarded as the most important policy and managerial issues, in a way that reflected their organisation’s role in the wider health economy and their own role in governance activities outside board meetings. We found no evidence of NEDs straying into an executive role. The pattern of NED behaviours was on balance more indicative of an active, strategic approach to governance than of passive monitoring or ‘rubber-stamping’.

These findings are informative in four ways. First, many NEDs in the US third sector regard their role as more honorific than practical [37]. The NHS NEDs that we studied were more active and assertive than that. Second, NEDs in certain British corporations have been criticised for insufficiently challenging managers [38]. We have reported examples, in the NHS boards we observed, of NEDs sometimes challenging, albeit in measured ways, what managers were saying. However events such as the avoidable patient deaths and poor treatment at Mid-Staffordshire General Hospital [33] show that the NHS has no grounds for complacency on this score. Third, NEDs’ reliance on EDs to propose directions for future activity is a pattern that has withstood several NHS ‘reforms’, including policies intended to strengthen NEDs’ role. Last, Austin’s analytic categories can informatively be applied to the analysis of board meetings. However this study also highlights ways in which Austin’s analytical framework requires further development for that purpose, among them ways of bringing illocution (speech acts’ attitudinal, emotional and motivational expressiveness and effects) into the analysis of meetings; and a fuller explanation of how illocution mediates and/or moderates the relationships between locution and perlocution. The present study has applied a principal-agent model of governance but another future research question is how an Austinian framework might also be used in a more transactional analysis of how the boards of ‘public firms’ (not only government departments [5]) exercise governance.

Differences in the stakeholders which the different types of board represent, and in the different organisations’ functions within (say) a health system, imply different criteria of ‘efficient’ organisational, and in particular Board, performance [32]. We have also found that assumptions about boards and director’s roles drawn from other countries and sectors do not always apply to NHS boards; a finding relevant to the assumption, in current UK health policy, that public bodies should preferably imitate private corporations. NEDs of different kinds of organisation may therefore require correspondingly different knowledge and skills and if so, different recruitment criteria. Finally, it may also be necessary to supplement the use of a board including NEDs with other means of maintaining the public accountability of health system providers.

## Abbreviations

CCG: Clinical commissioning group
ED: Executive director
FT: Foundation trust
NED: Non executive director
NHS: National Health Service
PCT: Primary care trust
SGT: Self-governing acute trust
SHA: Strategic health suthority
UK: United Kingdom
